# Neoadjuvant rh-endostatin, docetaxel and epirubicin for breast cancer: efficacy and safety in a prospective, randomized, phase II study

**DOI:** 10.1186/1471-2407-13-248

**Published:** 2013-05-21

**Authors:** Jianghao Chen, Qing Yao, Dong Li, Juliang Zhang, Ting Wang, Ming Yu, Xiaodong Zhou, Yi Huan, Jing Wang, Ling Wang

**Affiliations:** 1 Department of Vascular and Endocrine Surgery, Xijing Hospital, Fourth Military Medical University, 17 Changle West Road, Xi'an 710032, China; 2 Department of Ultrasound, Xijing Hospital, Fourth Military Medical University, 17 Changle West Road, Xi'an 710032, China; 3 Department of Radiology, Xijing Hospital, Fourth Military Medical University, 17 Changle West Road, Xi'an 710032, China; 4 Department of Nuclear Medicine, Xijing Hospital, Fourth Military Medical University, 17 Changle West Road, Xi'an 710032, China

**Keywords:** Breast cancer, Recombinant human endostatin, Neoadjuvant chemotherapy, Clinical trial

## Abstract

**Background:**

Recombinant human endostatin (rh-endostatin) is a novel antiangiogenesis drug developed in China. Previous experiments have shown that rh-endostatin can inhibit the proliferation and migration of endothelial cells and some types of tumor cells. In this study, we evaluated the efficacy and safety profiles of combination therapy of rh-endostatin and neoadjuvant chemotherapy for breast cancer patients in a prospective, randomized, controlled, phase II trial.

**Methods:**

Sixty-eight patients with core-biopsy confirmed breast cancer were allocated randomly to two groups to receive 3 cycles of intravenous administration of either neoadjuvant DE (docetaxel: 75 mg/m^2^, d1, epirubicin: 75 mg/m^2^, d1, every 3 weeks), or neoadjuvant DE combined with rh-endostatin (7.5 mg/m^2^, d1-d14, every 3 weeks). The primary end point was clinical response based upon Response Evaluation Criteria in Solid Tumors, and the secondary end point was safety and quality of life.

**Results:**

All patients were assessable for toxicity and 64 (94.2%) were assessable for efficacy evaluation. The objective response rate was 67.7% for chemotherapy (n = 31) and 90.9% for rh-endostatin plus chemotherapy (n = 33) (*P* = 0.021). A retrospective subset analysis revealed that rh-endostatin was more effective in premenopausal patients and patients with ECOG score of zero (*P* = 0.002 and *P* = 0.049, respectively). Five patients in the rh-endostatin plus chemotherapy arm achieved pathologic complete response compared with 2 in the chemotherapy arm (*P* = 0.428). No significant difference was identified in quality of life score and side effects (*P* > 0.05).

**Conclusion:**

The combination of rh-endostatin with chemotherapy produced a higher tumor response rate without increasing toxicity in breast cancer patients.

**Trial registration:**

ClinicalTrials.gov Identifier, NCT00604435

## Background

Breast cancer is one of the most common malignancies in women and its incidence continues to increase [1]. In the past 30 years, an improved understanding of the biological behavior of breast cancer has led to advancements in treatment.

Antiangiogenic therapy for cancer has attracted considerable attention. The rationale behind it is that tumor growth is dependent on angiogenesis [2], which was proposed by Dr. Folkman and has been confirmed by basic and clinical research. In 1997, O’Reilly *et al.*[3] isolated a peptide with a molecular weight of about 20 kD from murine hemangioendothelioma. By amino acid sequencing, this peptide was identified as a fragment of C-terminal type XVIII collagen with anti-angiogenic effects and was named endostatin. Recent studies indicated that endostatin was one of the most effective angiogenesis inhibitors currently known. Endostatin can directly target capillary endothelial cells around the tumor without detectable toxicity in normal cells. It may also inhibit cell migration, induce cell apoptosis, and play a multitargeted antiangiogenic role by regulating expression of vascular endothelial growth factor (VEGF) and activity of proteolytic enzymes, indirectly leading to the quiescence or reduction of tumors [4].

Recombinant human endostatin (rh-endostatin) is a new protein modified by an additional nine-amino acid sequence to the N-terminal of endostatin. Preclinical study indicated that rh-endostatin could inhibit tumor endothelial cell proliferation, angiogenesis and tumor growth. In 2001, rh-endostatin was approved by the State Food and Drug Administration of China (Approval No.: 2001SL029) for clinical research. From 2003 to 2004, Sun *et al.*[5] conducted a randomized, double-blind, multicenter, phase III clinical trial comparing treatment of vinorelbine and cisplatin (NP) plus rh-endostatin and NP alone in non-small cell lung cancer (NSCLC) patients. The objective response rate (ORR) was 35.4% and 19.5% (*P* < 0.01), the median time to progression was 6.3 and 3.6 months (*P* < 0.001), the median survival time was 14.8 and 9.9 months (*P* < 0.001), and the 1-year survival rate was 62.7% and 31.5% (*P* < 0.001) in NP plus rh-endostatin group and NP alone group, respectively. These data indicated that rh-endostatin had synergistic effects with NP. Rh-endostatin not only increased tumor response rate, but also significantly improved the overall survival (OS) without increasing the adverse effects. In another multicenter, randomized, double-blind, placebo-controlled study published in 2011, treatment with paclitaxel and carboplatin (TC) plus rh-endostatin improved ORR in patients with advanced NSCLC and exhibited a good safety profile, although the differences in progression free survival (PFS) or OS were not statistically significant from TC alone [6].

All these findings suggest that rh-endostatin may be effective as well in the treatment of other solid tumors, including breast cancer. To test this hypothesis, we designed a prospective, randomized, controlled phase II clinical trial to determine the efficacy and safety of neoadjuvant docetaxel and epirubicin (DE) with or without rh-endostatin for breast cancer patients (ClinicalTrials.gov Identifier: NCT00604435). It is the first registered clinical trial gauging the impact of rh-endostatin on breast cancer therapy.

## Methods

### Study design

This study was a prospective, randomized, parallel controlled, phase II trial. The primary end point was clinical and pathological response, and the secondary end point was safety and quality of life (QOL). This study was approved by the Ethics Committee of Fourth Military Medical University to be in accordance with the Declaration of Helsinki. Written informed consent was obtained from all participants before enrollment.

The inclusion criteria were (1) female, aged 18–70 years, (2) histologically confirmed invasive breast cancer (core needle biopsy for breast cancer diagnosis and fine needle aspiration for lymph node metastasis diagnosis), stage IIA to IIIC, and to be treated with primary systemic therapy, (3) without previous treatment, (4) Eastern Cooperative Oncology Group (ECOG) performance status 0–2, (5) normal hematologic function, (6) left ventricular ejection fraction greater than 50%, and (7) adequate liver or kidney function tests. Exclusion criteria included (1) allergic constitution or possible allergic reaction to drugs to be used in this study, (2) any concurrent uncontrolled medical or psychiatric disorder, (3) history of severe heart diseases, including congestive heart failure, unstable angina, uncontrolled arrhythmia, myocardial infarction, uncontrolled high blood pressure, or heart valve disease, (4) history of bleeding diathesis, and (5) being pregnant or nursing.

### Treatment schemes

All patients were prospectively registered in our central research database. Patients were randomly assigned to receive either 3 cycles of neoadjuvant DE (docetaxel: 75 mg/m^2^, d1, epirubicin: 75 mg/m^2^, d1, every 3 weeks), or 3 cycles of neoadjuvant DE and rh-endostatin (7.5 mg/m^2^, d1-d14, every 3 weeks) administered intravenously. Docetaxel was produced by Sanofi-Aventis Company, epirubicin by Pfizer Company, and rh-endostatin by Simcere-Medgenn Bio-Pharmaceuticals Company (National Medicine Permit No. S20050088).

### Clinical evaluation

At the time of diagnosis and after each cycle of therapy, the sizes of breast tumors and lymph nodes were measured, respectively, with physical examination and ultrasound [7]. The clinical response was classified as follows, according to Response Evaluation Criteria in Solid Tumors (RECIST) version 1.0 [8]: complete response (CR), disappearance of all target lesions; partial response (PR), at least a 30% decrease in the sum of the longest diameter of target lesions; progressive disease (PD): at least a 20% increase in the sum of the longest diameter of target lesions or the appearance of one or more new lesions; stable disease (SD): neither sufficient shrinkage to qualify for PR nor sufficient increase to qualify for PD. Tumor classified as CR or PR was defined as objective response (OR), and that classified as SD or PD was defined as no response (NR).

QOL was evaluated using the European Organization for Research and Treatment of Cancer Quality of Life of Questionnaire (EORTC QLQC30) questionnaire [9] at study entry, and prior to surgery. The adverse events during neoadjuvant therapy were graded based on the National Cancer Institute Common Terminology Criteria for Adverse Events (NCI CTCAE) version 4.03 [10].

### Surgery and adjuvant therapy

Following the completion of neoadjuvant therapy, all patients underwent Patey’s radical mastectomy or lumpectomy plus axillary clearance and then received 3 to 6 cycles of adjuvant chemotherapy. The number of cycles was decided in terms of clinical staging, histological grade, clinical and pathological response, apoptosis index, and biological markers such as hormonal receptor status, Ki-67 index, and human epidermal growth factor receptor 2 (HER2) expression. Radiotherapy was given to most (87%) patients, including all breast conservative cases. Patients with positive estrogen receptor (ER) and/or progesterone receptor (PR) received 5 years of endocrine therapy. All participants were followed up at 6-month intervals in an outpatient clinic.

### Tissue processing

Tissue samples, collected from biopsy and surgery, were fixed in 4% neutral formalin for 24 h and then embedded in paraffin blocks. Sections were cut at 3 μm and mounted on glass slides overnight at room temperature. Histological classification and grading were made using light microscopy examination of tissue sections stained with H&E. Pathological response of breast tumor was defined as follows: (a) complete response (pCR), no residual viable invasive tumor, ie, only *in situ* disease or tumor stroma remained; and (b) nonresponse, any viable tumor [11]. The status of hormone receptor and HER2 was examined by immunohistochemistry using a standard avidin-biotin complex technique [12,13].

### Statistical analysis

SPSS 11.0 for windows was used for statistical analysis. Normal distribution data were represented by mean ± standard deviation. Student’s *t* test was used to compare the mean after checking the homogeneity of variance. Enumeration data was compared using x^2^ test, and Fisher’s exact test was used for that with few cases. *P* < 0.05 was considered statistically significant.

## Results

### Baseline characteristics

From February 2008 to March 2010, 70 patients were enrolled in this study. Two of them failed to complete the first cycle of chemotherapy (one for economic reason and the other for uncontrollable hyperglycemia) and were excluded. Sixty-eight patients were assessable for toxicity and QOL evaluation and were randomly assigned to two groups. Two patients in each group failed to complete three cycles of primary systemic therapy (2 asked surgery ahead of schedule and 2 refused to continue chemotherapy), leaving 64 assessable for efficacy evaluation with 31 in DE group and 33 in DE plus rh-endostatin group.

The baseline characteristics of the study population including age, menopausal status, hormone receptor status, HER2 status, ECOG performance status, histology, axillary lymph node status, and clinical stage are given in Table 1. All these factors were well balanced between two groups (*P* > 0.05 for all comparison).

**Table 1:** Baseline characteristics (n = 64)

**Characteristic**	**DE + rh-endostatin** **(n = 33)** **n (%)**	**DE** **(n = 31)** **n (%)**
Age, years		
Median (range)	51.5 (33–68)	51.3 (35–67)
Menopausal status		
Premenopausal	14 (42.4)	13 (41.9)
Postmenopausal	18 (54.5)	14 (45.2)
Perimenopausal	1 (3.0)	4 (12.9)
ER status		
Positive	19 (57.6)	15 (48.4)
Negative	12 (36.4)	14 (45.2)
Unknown	2 (6.1)	2 (6.5)
PR status		
Positive	15 (45.5)	14 (45.2)
Negative	16 (48.5)	15 (48.4)
Unknown	2 (6.1)	2 (6.5)
HER2 status		
Positive	6 (18.2)	7 (22.6)
Negative	24 (72.7)	20 (64.5)
Unknown	3 (9.1)	4 (12.9)
ECOG performance status		
0	18 (54.5)	21 (67.7)
1	12 (36.4)	8 (25.8)
2	3 (9.1)	2 (6.5)
Histology		
Ductal	29 (87.9)	27 (80.6)
Lobular	2 (6.1)	3 (9.7)
Others	2 (6.1)	1 (3.2)
No. of metastatic lymph nodes		
0	16 (48.5)	12 (38.7)
1-3	6 (18.2)	11 (35.5)
4-9	7 (21.2)	5 (16.1)
≥10	4 (12.1)	3 (9.7)
TNM stage		
II	22 (66.7)	19 (61.3)
III	11 (33.3)	12 (38.7)

Abbreviations: *DE*, docetaxel and epirubicin; *ER*, estrogen receptor; *PR*, progesterone receptor; *HER2*, human epidermal growth factor receptor 2; *ECOG*, Eastern Cooperative Oncology Group.

### Clinical and pathologic response

Of the 33 patients in DE plus rh-endostatin group, 4 (12.1%) achieved CR, 26 (78.8%) achieved PR, 1 (3.0%) exhibited SD, and 2 (6.1%) exhibited PD. Of the 31 patients in the chemotherapy alone group, 2 (6.5%) achieved CR, 19 (61.3%) PR, 9 (29.0%) SD, and 1 (3.2%) PD. The ORR in patients receiving chemotherapy with or without rh-endostatin were 90.9% (30/33) and 67.7% (21/31), respectively, with significant difference (x^2^ = 5.300, *P* = 0.021).

pCR was identified in 7 (10.9%) of all the 64 patients with 5 (15.2%) in the rh-endostatin plus chemotherapy arm and 2 (6.5%) in the chemotherapy alone arm. A higher pCR rate was achieved following the combination therapy, but without significant difference from chemotherapy alone (*P* = 0.428).

### Outcome-influencing factor stratification

All the factors probably influencing the clinical response were stratified and analyzed. The factors included age, menopausal status, hormone receptor status, HER2 status, ECOG performance status, histology, axillary lymph node status, and clinical stage. No correlation was identified between ORR and age, hormone receptor status, HER2 status, histology, lymph node status or clinical stage (*P* > 0.05 for all). However, premenopausal women and those with ECOG score of zero demonstrated a significantly higher ORR (100% *vs* 46.3%, *P* = 0.002, and 94.4% *vs* 66.7%, *P* = 0.049, respectively). The details of stratification analysis are listed in Table 2.

**Table 2:** Stratification analysis of factors influencing outcomes

**Factors**	**DE + rh-endostatin** **(n = 33)** **OR (%)**	**DE** **(n = 31)** **OR (%)**	***p*value**
Age, years			
30-40	4 (100.0)	1 (100.0)	1.0
40-60	23 (92.0)	19 (76.0)	0.247
>60	3 (75.0)	1 (20.0)	0.206
Menopausal status			
Premenopausal	14 (100.0)	6 (46.2)	0.002
Postmenopausal	15 (83.3)	11 (78.6)	1.0
ER status			
Positive	17 (89.5)	11 (73.3)	0.210
Negative	11 (91.7)	9 (64.3)	0.590
PR status			
Positive	14 (93.3)	9 (64.3)	0.080
Negative	14 (87.5)	11 (73.3)	0.394
HER2 status			
Positive	6 (100.0)	6 (85.7)	1.0
Negative	22 (91.7)	13 (65.0)	0.160
ECOG performance status			
0	17 (94.4)	14 (66.7)	0.049
1	10 (83.3)	5 (62.5)	0.347
2	3 (100.0)	2 (100.0)	1.0
Histology			
Ductal	26 (89.7)	19 (70.4)	0.096
Lobular and others	4 (100.0)	2 (50.0)	0.206
No. of metastatic lymph nodes			
0	16 (100.0)	10 (83.3)	0.175
1-3	4 (66.7)	4 (36.4)	0.335
4-9	6 (85.7)	4 (80.0)	1.0
≥10	4 (100.0)	3 (100.0)	1.0
TNM stage			
II	20 (90.9)	13 (68.4)	0.219
III	10 (90.9)	8 (66.7)	0.317

Abbreviations: *DE*, docetaxel and epirubicin; *ER*, estrogen receptor; *PR*, progesterone receptor; *HER2*, human epidermal growth factor receptor 2; *ECOG*, Eastern Cooperative Oncology Group.

### Quality of life

Sixty-eight patients completed at least one cycle of treatment and received QOL assessment (Table 3). No significant difference was found between the two groups either before or after treatment (*P* > 0.05), indicating that rh-endostatin may exert little effect upon QOL of patients.

**Table 3:** Comparison of quality of life

**QOL score**	**n**	**Pretreatment**	**Posttreatment**
DE + rh-endostatin	35	53.12 ± 3.43	53.18 ± 4.41
DE	33	53.55 ± 4.87	51.76 ± 5.43

Abbreviations: *QOL*, quality of life; *DE*, docetaxel and epirubicin.

### Adverse events

Sixty-eight patients were assessable for toxicity evaluation (Table 4). The total incidence of adverse events was 81.2% and 79.3%, respectively, in the rh-endostatin plus chemotherapy arm and chemotherapy alone arm. Most of the adverse events were of grade 1 and 2. Grade 3 and 4 adverse events included leucopenia, neutropenia, nausea, and vomiting. No significant difference was found between the two groups, either in the incidence of overall adverse events or in the incidence of grade 3/4 adverse events (*P* > 0.05 for all).

**Table 4:** Comparison of adverse events

**Adverse event**	**DE + rh-endostatin (n = 35)**		**DE (n = 33)**	
	**Grade 1-4**	**Grade 3-4**	**Grade 1-4**	**Grade 3-4**
	**n (%)**	**n (%)**	**n (%)**	**n (%)**
Anemia	9 (25.7)	1 (2.9)	8 (24.2)	2 (6.1)
Leukopenia	18 (51.4)	7 (20.0)	17 (51.5)	9 (27.3)
Neutropenia	19 (54.3)	7 (20.0)	16 (48.5)	8 (24.2)
Thrombocytopenia	3 (8.6)	0 (0)	4 (12.1)	0 (0)
Bilirubin elevation	4 (11.4)	1 (2.9)	1 (3.0)	0 (0)
Transaminase elevation	1 (2.9)	0 (0)	2 (6.1)	0 (0)
Stomatitis	0 (0)	0 (0)	2 (6.1)	0 (0)
Nausea/vomiting	20 (57.1)	10 (28.6)	23 (69.7)	13 (39.4)
Diarrhea	1 (2.9)	0 (0)	0 (0)	0 (0)
Astriction	0 (0)	0 (0)	1 (3.0)	0 (0)
Proteinuria	3 (8.6)	0 (0)	1 (3.0)	0 (0)
Alopecia	23 (65.7)		22 (66.7)	
Arhythmia	2 (5.7)	0 (0)	2 (6.1)	0 (0)
Cardiovascular dysfunction	3 (8.6)	0 (0)	2 (6.1)	0 (0)
Peripheral neuropathy	2 (5.7)	0 (0)	1 (3.0)	0 (0)
Hand-foot syndrome	4 (11.4)	3 (8.6)	5 (15.2)	1 (3.0)
Fatigue	26 (74.3)	1 (2.9)	25 (75.8)	2 (6.1)

Abbreviation: *DE*, docetaxel and epirubicin.

## Discussion

Neoadjuvant chemotherapy, also known as preoperative or primary chemotherapy, is widely used in the management of breast cancer patients to treat occult systemic disease, reduce the tumor bulk, and increase the likelihood of breast conservation, while not affecting the local recurrence hazard [14,15], or compromising survival [16]. In the mean time, preoperative therapy provides an opportunity to gain more insight into the cellular and molecular changes involved in tumor response.

The current systemic treatment of breast cancer has been developed rapidly in the past 10 years. Compared with conventional cytotoxic chemotherapy, the systemic treatment is more sophisticated and specific, characterized by multiple cancer targets. One promising strategy is targeting the proangiogenic VEGF, either by ligand sequestration (preventing VEGF receptor binding) or inhibiting downstream receptor signaling [17]. Thus far, more than 30 kinds of antiangiogenesis agents have been approved for clinical practice or ongoing preclinical and clinical trials.

Bevacizumab (Avastin), a recombinant humanized antibody against VEGF, is the first antiangiogenesis drug approved for clinical practice. A recent report of a pooled analyses of metastatic breast cancer (MBC) patients receiving bevacizumab-based therapy showed that the addition of bevacizumab significantly prolonged PFS time [17]. Although the PFS interval might depend on the evaluation methods and schedules used, the PFS as a study endpoint currently represents the most sensitive parameter to assess the efficacy of an experimental drug in metastatic disease, especially when a longer PFS duration is associated with a higher ORR or a measurable improvement in QOL. The most common adverse effects of bevacizumab include headache, nausea, vomiting, anorexia, stomatitis, constipation, upper-respiratory-tract infection, epistaxis, dyspnea, and proteinuria. The most serious adverse effects include hypertensive crisis, nephritic syndrome, hemorrhage, gastrointestinal perforation, wound-healing complications, and congestive heart failure [18].

Rh-endostatin (Endostar) is a new recombinant humanized endostatin expressed and purified in E. coli. The additional nine-amino acid sequence to the N-terminal of endostatin may effectively simplify purification and improve the stability of the protein [19,20], although the exact mechanism of rh-endostatin as antiangiogenesis drug remains unclear. Previous studies showed that endostatin may downregulates many signaling pathways in human microvascular endothelium associated with proangiogenic activity, and simultaneously upregulate many antiangiogenic genes [21,22]. It was also found that endostatin could lower VEGF expression in colon and cervical cancer [23,24]. Based on the positive data from a phase III trial [5], China State Food and Drug Administration approved rh-endostatin plus NP as a first line therapy for advanced NSCLC in 2005.

Angiogenesis is an important step in the proliferation of breast cancer cells and is thought to precede invasive disease [18]. In situ hybridization revealed high levels of VEGF in ductal carcinoma in situ, infiltrating ductal carcinoma, and metastatic ductal carcinoma [25], further rationalizing the addition of rh-endostatin for breast cancer therapy. In this study, 68 patients with stage II or III primary breast cancer were allocated randomly to two groups to receive either 3 cycles of neoadjuvant DE, or 3 cycles of neoadjuvant DE plus rh-endostatin. The primary end point was to determine the impact of the addition of rh-endostatin on the clinical and pathologic response. Among the 64 patients assessable for efficacy evaluation, the ORR was 90.9% for patients treated with rh-endostatin plus chemotherapy compared with 67.7% for patients treated with chemotherapy alone, indicating that the combination of rh-endostatin with chemotherapy may effectively improve the chance of tumor downstage before surgery. Follow-up study of the patients will provide data as to whether the increased ORR rate can be translated into high PFS and OS. Stratification analysis revealed that premenopausal patients and patients with ECOG sore of zero had higher ORR, indicating that premenopausal women with good performance status may benefit more from rh-endostatin combination therapy.

For patients treated with anthracycline-based neoadjuvant chemotherapy, a higher pCR has been shown to be predictive of improved PFS and OS [16,26]. In this study, the combination of rh-endostatin with chemotherapy achieved a higher pCR rate (15.2%), but not statistically different from chemotherapy alone (6.5%), which is probably due to the small number of patients enrolled in the current trail. In fact, we are designing a multicenter, prospective, randomized, controlled phase III clinical trial that will include a total of 800 patients to further evaluate the efficacy and safety of rh-endostatin in breast cancer treatment (NCT01479036).

In the present study, the total incidence of adverse events was 81.2% in the rh-endostatin plus chemotherapy group and 79.3% in the chemotherapy alone group. Most of the adverse events were of grade 1 or 2. Grade 3/4 adverse events associated with rh-endostatin included leucopenia, neutropenia, nausea, vomiting, and hand-foot syndrome. Statistical analysis indicated that the addition of rh-endostatin did not increase drug-related toxicities, especially those typically observed in other antiangiogenesis agents such as bevacizumab. A systematic review and meta-analysis reported that the addition of bevacizumab to chemotherapy in MBC patients did increase the risk of left ventricular dysfunction and hemorrhagic events, whereas it was not associated with a significant increase in grade ≥3 arterial or venous thromboembolic events, gastrointestinal perforation, or fatal events [27]. In addition, rh-endostatin exhibited good tolerance, as verified in QOL score questionnaire before and after treatment. However, it should be noted that patients with history of serious heart disease, and with liver or kidney dysfunction were excluded from the trial. The impact of rh-endostatin upon the incidence of adverse events should be further assessed in larger scale trials in future.

Some limitations of the present study have to be considered. Since there is no previous report on rh-endostatin in breast cancer therapy at the neoadjuvant setting, we did not make a pre-planned power calculation. Second, the number of patients enrolled is relatively small, making a multiple factor analysis inappropriate. Thus, we must state here that the subset analyses used in this study were mostly exploratory.

## Conclusions

This prospective, randomized, controlled phase II clinical trial demonstrated that the combination of rh-endostatin and DE chemotherapy may improve overall clinical response in patients with stage II or III breast cancer, especially pre-menopausal patients and those having ECOG score of zero, without increasing adverse events. Therefore rh-endostatin can be used as a safe and effective strategy in neoadjuvant treatment of breast cancer.

## Abbreviations

VEGF: Vascular endothelial growth factor; rh-endostatin: Recombinant human endostatin; NP: Vinorelbine and cisplatin; NSCLC: Non-small cell lung cancer; ORR: Objective response rate; OS: Overall survival; TC: Paclitaxel and carboplatin; PFS: Progression free survival; DE: Docetaxel and epirubicin; QOL: Quality of life; ECOG: Eastern Cooperative Oncology Group; RECIST: Response Evaluation Criteria in Solid Tumors; CR: Complete response; PR: Partial response; PD: Progressive disease; SD: Stable disease; OR: Objective response; NR: No response; EORTC QLQC30: European Organization for Research and Treatment of Cancer Quality of Life of Questionnaire; NCI CTCAE: National Cancer Institute Common Terminology Criteria for Adverse Events; HER2: Human epidermal growth factor receptor 2; ER: Estrogen receptor; PR: Progesterone receptor; pCR: Pathological complete response; MBC: Metastatic breast cancer.

## Competing interests

The authors declare that they have no competing interests.

## Authors’ contributions

JC preformed core biopsies, participated in study design, and drafted the manuscript. QY carried out pathologic and immunohistochemical examination. DL is an independent statistician and was responsible for the statistical analysis and data interpretation. JZ and TW collected clinical data. MY and XZ performed fine needle aspiration and lymph node response evaluation. YH and JW performed radiological examination. LW conceived the study and helped finalize the manuscript. All authors read and approved the final manuscript.

## Pre-publication history

The pre-publication history for this paper can be accessed here:

http://www.biomedcentral.com/1471-2407/13/248/prepub

## References

[B1] Parkin DM Pisani P Ferlay J Estimates of the worldwide incidence of 25 major cancers in 1999 Int J Cancer 1999 80 827 841 10074914 10.1002/(sici)1097-0215(19990315)80:6<827::aid-ijc6>3.0.co;2-p

[B2] Folkman J Tumor angiogenesis: therapeutic implications N Engl J Med 1971 285 1182 1186 10.1056/NEJM197111182852108 4938153

[B3] O'Reilly MS Boehm T Shing Y Fukai N Vasios G Lane WS Flynn E Birkhead JR Olsen BR Folkman J Endostatin: an endogenous inhibitor of angiogenesis and tumor growth Cell 1997 88 277 285 10.1016/S0092-8674(00)81848-6 9008168

[B4] Folkman J Antiangiogenesis in cancer therapy-endostatin and its mechanisms of action Exp Cell Res 2006 312 594 607 10.1016/j.yexcr.2005.11.015 16376330

[B5] Wang J Sun Y Liu Y Yu Q Zhang Y Li K Zhu Y Zhou Q Hou M Guan Z Li W Zhuang W Wang D Liang H Qin F Lu H Liu X Sun H Zhang Y Wang J Luo S Yang R Tu Y Wang X Song S Zhou J You L Wang J Yao C Results of randomized, multicenter, double-blind phase III trial of rh-endostatin (YH-16) in treatment of advanced non-small cell lung cancer patients Zhongguo Fei Ai Za Zhi 2005 8 283 290 21108883 10.3779/j.issn.1009-3419.2005.04.07

[B6] Han B Xiu Q Wang H Shen J Gu A Luo Y Bai C Guo S Liu W Zhuang Z Zhang Y Zhao Y Jiang L Zhou J Jin X A multicenter, randomized, double-blind, placebo-controlled study to evaluate the efficacy of paclitaxel-carboplatin alone or with endostar for advanced non-small cell lung cancer J Thorac Oncol 2011 6 1104 1109 10.1097/JTO.0b013e3182166b6b 21532504

[B7] Herrada J Iyer RB Atkinson EN Sneige N Buzdar AU Hortobagyi GN Relative value of physical examination, mammography, and breast sonography in evaluating the size of the primary tumor and regional lymph node metastases in women receiving neoadjuvant chemotherapy for locally advanced breast carcinoma Clin Cancer Res 1997 3 1565 1569 9815844

[B8] Therasse P Arbuck SG Eisenhauer EA Wanders J Kaplan RS Rubinstein L Verweij J Van Glabbeke M van Oosterom AT Christian MC Gwyther SG New guidelines to evaluate the response to treatment in solid tumors. European Organization for Research and Treatment of Cancer, National Cancer Institute of the United States, National Cancer Institute of Canada J Natl Cancer Inst 2000 92 205 216 10.1093/jnci/92.3.205 10655437

[B9] Apolone G Filiberti A Cifani S Ruggiata R Mosconi P Evaluation of the EORTC QLQ-C30 questionnaire: a comparison with SF-36 Health Survey in a cohort of Italian long-survival cancer patients Ann Oncol 1998 9 549 557 10.1023/A:1008264412398 9653497

[B10] National Cancer Institute Cancer Therapy Evaluation Program. US National Institutes of Health Common Terminology Criteria for Adverse Events (CTCAE), v4.03: June 14, 2010 Available at: http://evs.nci.nih.gov/ftp1/CTCAE/About.html

[B11] Kurosumi M Significance and problems in evaluations of pathological responses to neoadjuvant therapy for breast cancer Breast Cancer 2006 13 254 259 10.2325/jbcs.13.254 16929118

[B12] Saccani Jotti G Johnston SR Salter J Detre S Dowsett M Comparison of new immunohistochemical assay for oestrogen receptor in paraffin wax embedded breast carcinoma tissue with quantitative enzyme immunoassay J Clin Pathol 1994 47 900 905 10.1136/jcp.47.10.900 7962603 PMC502173

[B13] Gusterson BA Gelber RD Goldhirsch A Price KN Säve-Söderborgh J Anbazhagan R Styles J Rudenstam CM Golouh R Reed R Prognostic importance of c-erbB-2 expression in breast cancer. International (Ludwig) Breast Cancer Study Group J Clin Oncol 1992 10 1049 1056 1351538 10.1200/JCO.1992.10.7.1049

[B14] Powles TJ Hickish TF Makris A Ashley SE O'Brien ME Tidy VA Casey S Nash AG Sacks N Cosgrove D Randomized trial of chemoendocrine therapy started before or after surgery for treatment of primary breast cancer J Clin Oncol 1995 13 547 552 7884414 10.1200/JCO.1995.13.3.547

[B15] Smith IE Walsh G Jones A Prendiville J Johnston S Gusterson B Ramage F Robertshaw H Sacks N Ebbs S High complete remission rates with primary neoadjuvant infusional chemotherapy for large early breast cancer J Clin Oncol 1995 13 424 429 7844604 10.1200/JCO.1995.13.2.424

[B16] Fisher B Bryant J Wolmark N Mamounas E Brown A Fisher ER Wickerham DL Begovic M DeCillis A Robidoux A Margolese RG Cruz AB Jr Hoehn JL Lees AW Dimitrov NV Bear HD Effect of preoperative chemotherapy on the outcome of women with operable breast cancer J Clin Oncol 1998 16 2672 2685 9704717 10.1200/JCO.1998.16.8.2672

[B17] Alvarez RH Guarneri V Icli F Johnston S Khayat D Loibl S Martin M Zielinski C Conte P Hortobagyi GN Bevacizumab treatment for advanced breast cancer Oncologist 2011 16 1684 1697 10.1634/theoncologist.2011-0113 21976315 PMC3248767

[B18] Motl S Bevacizumab in combination chemotherapy for colorectal and other cancers Am J Health Syst Pharm 2005 62 1021 1032 15901587 10.1093/ajhp/62.10.1021

[B19] Ling Y Yang Y Lu N You QD Wang S Gao Y Chen Y Guo QL Endostar, a novel recombinant human endostatin, exerts antiangiogenic effect via blocking VEGF-induced tyrosine phosphorylation of KDR/Flk-1 of endothelial cells Biochem Biophys Res Commun 2007 361 79 84 10.1016/j.bbrc.2007.06.155 17644065

[B20] Song HF Liu XW Zhang HN Zhu BZ Yuan SJ Liu SY Tang ZM Pharmacokinetics of His-tag recombinant human endostatin in Rhesus monkeys Acta Pharmacol Sin 2005 26 124 128 10.1111/j.1745-7254.2005.00009.x 15659125

[B21] Benezra R Rafii S Endostatin's endpoints-Deciphering the endostatin antiangiogenic pathway Cancer Cell 2004 5 205 206 10.1016/S1535-6108(04)00057-1 15050911

[B22] Abdollahi A Hahnfeldt P Maercker C Gröne HJ Debus J Ansorge W Folkman J Hlatky L Huber PE Endostatin's antiangiogenic signaling network Mol Cell 2004 13 649 663 10.1016/S1097-2765(04)00102-9 15023336

[B23] Jia Y Liu M Huang W Wang Z He Y Wu J Ren S Ju Y Geng R Li Z Recombinant human endostatin Endostar inhibits tumor growth and metastasis in a mouse xenograft model of colon cancer Pathol Oncol Res 2012 18 315 323 10.1007/s12253-011-9447-y 21938482 PMC3313035

[B24] Jia Y Liu M Cao L Zhao X Wu J Lu F Li Y He Y Ren S Ju Y Wang Y Li Z Recombinant human endostatin, Endostar, enhances the effects of chemo-radiotherapy in a mouse cervical cancer xenograft model Eur J Gynaecol Oncol 2011 32 316 324 21797125

[B25] Brown LF Berse B Jackman RW Tognazzi K Guidi AJ Dvorak HF Senger DR Connolly JL Schnitt SJ Expression of vascular permeability factor (vascular endothelial growth factor) and its receptors in breast cancer Hum Pathol 1995 26 86 91 10.1016/0046-8177(95)90119-1 7821921

[B26] Kuerer HM Newman LA Smith TL Ames FC Hunt KK Dhingra K Theriault RL Singh G Binkley SM Sneige N Buchholz TA Ross MI McNeese MD Buzdar AU Hortobagyi GN Singletary SE Clinical course of breast cancer patients with complete pathologic primary tumor and axillary lymph node response to doxorubicin-based neoadjuvant chemotherapy J Clin Oncol 1999 17 460 469 10080586 10.1200/JCO.1999.17.2.460

[B27] Cortes J Calvo V Ramírez-Merino N O'Shaughnessy J Brufsky A Robert N Vidal M Muñoz E Perez J Dawood S Saura C Di Cosimo S González-Martín A Bellet M Silva OE Miles D Llombart A Baselga J Adverse events risk associated with bevacizumab addition to breast cancer chemotherapy: a meta-analysis Ann Oncol 2012 23 1130 1137 10.1093/annonc/mdr432 21976387

